# Ageing Population's Impact on Economic Growth in Malaysia From 1981 to 2019: Evidence From an Autoregressive Distributed Lag Approach

**DOI:** 10.3389/fpubh.2021.731554

**Published:** 2021-11-24

**Authors:** Siti Nur Ain Mohd, Ayunee Anis Ishak, Doris Padmini Selvaratnam

**Affiliations:** Faculty of Economics and Management, Universiti Kebangsaan, Bangi, Malaysia

**Keywords:** ageing population, economic growth, endogenous growth model, ARDL, Malaysia

## Abstract

This study investigates the impact of the ageing population on the economic growth for short- and long-run estimations in Malaysia, by using time series data from 1981 to 2019. This study adopts the autoregressive distributed lag (ARDL) method with the Bound test approach for the long-run estimation and the vector error correction model for the short-run estimation. Several econometric diagnostic tests were applied for validation and the appropriate model specification basis. The estimated result of this work indicates that the age dependency ratio proxy for the ageing population variable has a significant negative impact on economic growth in Malaysia. A 1% increase in old age dependency will decline gross domestic product's (GDP's) growth by an average of 6.6043% at the 5% level of significance. Hence, an increase in the ageing population will impede economic growth. Although controlled variables (e.g., physical capital, labour participation, and human capital) have a significant positive impact on economic growth in Malaysia, there is evidence of a long- and short-run relationship between economic growth and the ageing population variable, and also the control variable.

## Introduction

**Figure 1:**
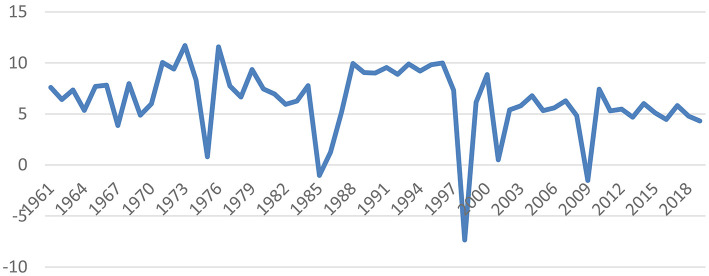
GDP growth in Malaysia, 1961–2019. Source: DOSM (2).

In general, there is a constant fluctuation in gross domestic products (GDP) in Malaysia over time. Figure 1 shows the GDP growth in Malaysia from 1961 to 2019. There was a sharp decline in 1985 due to low commodity prices. In 1998, the Asian Financial Crisis led to negative GDP growth. In 2009, the global financial crisis led to negative GDP growth. On the contrary, the increase in GDP for 1973 and 1976 was due to the increase in oil prices, as Malaysia is an oil exporter. In 2015, there was a reduction in GDP growth due to the goods and services tax (GST) implementation; therefore, the government provided Bantuan Rakyat 1 Malaysia to increase the consumption of citizens. In 2009, to reduce the impact of the global recession on Malaysians, the monetary policy of the overnight policy rate was reduced. Therefore, we can see that Malaysia used both fiscal and monetary approaches to reduce the impact of the recession. According to WHO (1)^1^, the continued decline in fertility rates followed by an increase in life expectancy will likely result in an increase in the ageing of the world's populations, where there is an increase of people above the age of 65 years compared with younger people. A nation is considered to have an ageing population if 7% of the population is 65 years old and above. Currently, Malaysia is moving towards having an ageing population. It is estimated that 7% of the population (2.3 million people) will be 65 years old and above in 2020 (2).

In Table 1, the age dependency ratio for older people shows an increasing trend, whereas the fertility rate in Malaysia shows a decreasing trend. Research done by Tang and Tey (6) in Malaysia found that higher education, career development of women, and the cost of living led to a decline in the fertility rate for Malaysia. Low fertility rates and increases in longevity can result in an increase in the aged population (7).

**Table 1:** The age dependency ratio, fertility rate, and government health expenditure (2000–2018).

**Year**	**Age dependency ratio**	**Fertility rate**	**Government health expenditure (% of GDP)**
2000	6.241	2.78	1.17
2002	6.527	2.54	1.37
2004	6.695	2.36	1.46
2006	6.967	2.25	1.67
2008	7.238	2.19	1.60
2010	7.359	2.15	1.68
2012	7.812	2.11	1.86
2014	8.373	2.07	2.03
2016	8.980	2.04	1.89
2018	9.623	2.00	1.92

*Source: World Bank (3–5)*.

Conversely, the general expenditure of the government on health is increasing slightly. According to Meijer et al. (8), the increase in health expenditure is due to the usage of advanced technology, which is likely to benefit Malaysians (including the elderly). Due to the increase of an ageing population in Malaysia, the fifth thrust in Tenth Malaysia Plan aims to focus on the elderly by encouraging the elderly to stay active, productive, and healthy as their age (9).

Both developed and developing countries (such as China, Thailand, Sri Lanka, etc.) experience an ageing population. However, it could create certain impacts for Malaysia. In Table 1, the population of people aged 65 years and above increased tremendously, whereas the growth of general expenditure for healthcare only increased slightly over the year. Meanwhile, Japan invested 10.9% of its GDP in 2018 for health expenditure, as it is one of the countries with a higher ageing population (10). Therefore, by moving towards an ageing population, the Malaysian government needs to increase budget allocation for public healthcare expenditure.

Furthermore, having an ageing population could impact the number of labour participants, as there is a decline in population growth. According to Otsu and Shibayama (7), population ageing will decrease the income of the country as it leads to a decline in the size of the workforce. An increase in the ageing population might also lead to a decline in the productivity of the workers. This can be supported by Börsch-Supan et al. (11), who found that older individuals will lead to less productive work outputs after a certain age. Therefore, an increase in the ageing population will likely slow down the productivity of a country.

Moreover, based on the research done by Maestas et al. (12)^2^, it was found that ageing will lead to a decrease in economic growth for the United States. Between 1980 and 2010, a 16.8% increase in the population who were 60 years and older led to a 9.2% reduction in GDP. Therefore, having an aged population might slow down Malaysia in becoming one of the developed countries of 2030. Lastly, it is important to have more GDP growth in the country as the income can be used by the government to provide more public goods to Malaysians. In view of the possible problems that may occur due to an ageing population, it is wise for the government to implement policies that help to solve the ageing population so that it can facilitate an increase in GDP growth. The limitation of the study is that there is a limited number of data available in Malaysia.

The study focuses on the GDP growth of Malaysia. The variables used are the age dependency ratio as a proxy to the ageing population, and control variables like physical capital, labour participation, human capital, and GDP growth for Malaysia. The data covers 39 years, 1981–2019. This study primarily investigates the impact of an ageing population on economic growth in Malaysia. The objectives of this study include an investigation into the relationships between the ageing population and control variables, such as capital and human capital with GDP growth, the size of the effect of each factor on the GDP growth during the short run, and identification of applicable policies to increase GDP growth in Malaysia.

This study attempts to answer the following research questions:

1. What are the determinants of GDP growth in Malaysia?
2. How can an ageing population, labour participation, physical capital, and human capital make an impact on GDP growth in the short- and long run?

## Literature Review

Several researchers have conducted research works aimed at studying the impact of the ageing population to recommend policy frameworks that will assist the relevant institutions (such as economic planning units and authorities) to provide essential policy instruments and social needs to the population. This will boost the economic growth and development process, especially in developing and underdeveloped countries. These theoretical and empirical studies are examined in this section.

### Theoretical Framework

From a theoretical framework, the economic theory argues that an ageing population can impede the growth of the economy. Modigliani and Brumberg (13), through their life cycle hypothesis, support the opinion that an ageing population can slow down economic growth. The life cycle hypothesis suggests that at an early stage, the ageing of the population will affect a rise in national savings. However, as the population continues to age and some relative proportion of the population reaches their retirement age, this hypothesis predicts a reduction in aggregate savings due to an increase in the ageing population.

Moreover, Solow's growth theory states that in an economy where the population is ageing, the stable economic growth of a country becomes difficult to attain. Stable growth conditions or steady state growth are only possible if the age structure of the population remains constant. However, an economy with an ageing population has an inconstant age structure. Hence, this is only possible during the transition of the economy towards its steady state. Based on this theory, the ageing of a population has a negative impact on economic growth (14, 15).

In addition, the Malthusian catastrophe by Thomas Malthus (16), in his book titled *An Essay on the Principle of Population*, describes a world in which uncontrolled population growth exceeds the resources needed to survive. He argued that human populations tend to grow at a much faster rate than human needs can grow, such as food, clothes, and other agriculture-based products. Therefore, Malthus (16) states that humanity is condemned to living in poverty forever because the growth of agricultural production will always be overtaken by population growth. Malthus's theory raises a question as to the recent argument about the consequences of an ageing population.

Romer (17), Grossman and Helpman (18), and Aghion and Howitt (19) state through endogenous growth models that the population size of a certain country is essential for the long-term development of the economy. The argument is that larger countries can grow more quickly because they have more scientists to employ and feature larger markets with more profit opportunities for competitive and innovative firms. Moreover, Romer (17) argues that a drop in both mortality and fertility lead to population ageing while leaving the population size constant. Therefore, our research will employ an endogenous growth model and focus on the implications of population ageing for GDP per capita growth. Since technological progress has been identified as the main driving force behind economic growth (17), we are particularly interested in seeing the effects of an ageing population on economic growth that will include labour, physical capital, and human capital as our control variables.

Prettner (20) combines endogenous growth models by Romer (17) with semiendogenous growth models and found that a rise in longevity has a positive impact on per economic growth. The reduction in fertility rates has a negative impact on economic growth. Furthermore, the positive longevity effect dominates the negative fertility effect. Population ageing fosters long-run growth in the endogenous growth framework. The main conclusion of Prettner (20) is that constant demographic change does not necessarily hinder technological progress and constitutes economic prosperity. He also argues that declining birth and death rates could lead to an increase in the rate of economic growth.

### Empirical Framework

Selvaratnam et al. (21) emphasise that the increase in life expectancy in Malaysia has led to an increase in government spending on reward and pension costs, as well as the employee's provident fund (EPF) and health expenditure for the elderly. An increase in the population life expectancy causes the older age group to significantly increase, which impacts the economic and social aspects. This presents a financial burden to individuals, families, and policymakers. It involves substantial expenses to provide housing and health facilities. Furthermore, Louria (22) emphasises that an increase in average life expectancy has the potential to increase longevity to about 100–120 years. This will create many social problems, such as a possible increase in health expenditure for the population aged 65+, quality of life issues, the potential for more financial resources, challenges to the possibility of social security and pensions, and other possible issues. By using the autoregressive distributed lag (ARDL) method and vector error correction model, Baharin and Saad (23) support these findings where rising numbers of the ageing population significantly affected health expenditure in Malaysia. Similar work by Ismail et al. (15) argues that the reduction of the old dependency ratio can reduce the tax and social security contributions paid by employees to fund retirement income and healthcare for the elderly. Therefore, it can also increase the labour supply. This means that there are fewer people to feed and thus more accumulated savings for productive investments in the economy. Therefore, an increase in the old age dependency ratio means that there is an increase in ageing that will hinder economic growth.

On an empirical level, the relationship between the ageing population and growth has been analysed in detail with various results. Certain literature found negative relationships between an ageing population and economic growth, such as the study by Brendan and Sek (24). By adopting the ARDL bound testing approach, they found a significant negative long-run relationship between dependency on the old and economic growth in India. This applies to India, which is expected to experience the negative effect of old age and risk a drop in saving rates and unemployment. From this study on the dependency on the old ratio, most countries such as South Korea, the Philippines, Thailand, Malaysia, and Singapore revealed a negative effect but an insignificant result. This indicates that most Asian countries have a higher youthful population compared with the ageing population, since individuals under the age of 15 contribute highly to the economy, more so than those aged 65 and above. Other studies by Maestas et al. (12)^3^ and Rahman (25) also found that the declining fertility rate and rising ageing proxies (such as the old age dependency ratio and population aged 65+) tend to slow down economic growth. However, Teixeira et al. (26) found that the ageing population negatively impacts the growth of developed countries, but not that of less developed countries or developing countries. They argue that the growth of the old age dependency ratio significantly reduces the growth prospects of developing countries, whereas growth of the ageing index and the old age dependency ratio significantly constrains the growth of less developing countries.

Some studies found positive relationships between ageing populations and economic growth, such as those highlighted by Brendan and Sek (24). They found that Japan has the highest old age dependency; thus it has long entered the phase of ageing. To overcome these problems, Japan has been using active ageing policies (such as the Silver Human Resource Centre) and various policies that employ the elderly into the workforce. This initiative will be reducing the negative effect of ageing and simultaneously creating a more positive effect on their economy. Other studies using ARDL by Ismail et al. (15) and Taasim (27) found that the age dependency ratio, population aged 65+, and a reduction of fertility have a positive impact on economic growth. Furthermore, Futagami and Nakajima (28) found positive effects of population ageing on economic growth, such as the development of labour-saving technology and increased investments into human capital. Scarth (29) argues that population ageing could lead to productivity growth by motivating increased investments into human capital, as labour becomes a relatively scarce production factor resource. For this reason, policymakers should focus on improvising policies on long-term care and on investments in technological advancements, education, and health.

## Methodology

The study was conducted to estimate the impact of the ageing population on economic growth in Malaysia. The data for the relevant variables were obtained from the World Bank and Penn World Table 9. The variables used for this analysis include the GDP growth per capita and age dependency ratio, the old population (% of working-age population), the control variables [i.e., gross capital formation (% of GDP) as a proxy to physical capital], labour force participation rate, total (% of total population ages 15+), and human capital index, based on the years of schooling and returns into education (15, 24–26). All the data for these variables were collected from 1981 to 2019, which covers a period of 39 years.

The theoretical framework model used in our study is based on Romer (17). It applies endogenous growth theory to claim that human capital is a significant determinant of economic growth. The equation is as follows:


$$\begin{array}{c} {\text{Y}}_{\text{t}}=\text{f }\left({\text{K}}_{\text{t}},{\text{L}}_{\text{t}},{\text{H}}_{\text{t}}\right), \end{array}$$

where *Y*_t_ is output, *K*_t_ is capital, *L*_t_ is labour force participation rate, and *H*_t_ is the human capital. This study was carried out by modifying the model developed by some researchers, such as Romer (17), Pretnerr (20), Ismail et al. (15), and Brendan and Sek (24). The specification of the economic growth model for this study is as follows:


$$\begin{array}{ccc} GDPG_{t}=\;B_{0}+{\;B_{1}LAGEING}_{t}+\;B_{2}LK_{t}+{\;B_{3}LL}_{t} & + & \;{\;B_{4}LHC}_{t}\\ + & \varepsilon_{t} \end{array}$$

where *GDPG*_*t*_ represents the included *GDP* growth per capita, acting as the economic growth variable to be described in the model. *AGEING*_*t*_ represents the age dependency ratio, old population (% of working-age population), and control variables *K*_*t*_ is the gross capital formation (% of GDP) as a proxy to physical capital. *L*_*t*_ is the labour force participation rate, total (% of total population ages 15+), and *HC*_*t*_ is the human capital index, based on years of schooling and returns to education, respectively.

In this research, the analysis was conducted by using the ARDL model approach. Before proceeding with the ARDL method, we conducted a pretest to check whether our data was stationary. We examined whether the data integrated at I (0), I (1), or I (2). If the data is not stationary at the same level, we can use ARDLs. In this study, we conduct the Philip–Perron (PP) test and Augmented Dickey Fuller (ADF) test. The primary focus of the analysis was to examine the long-run relationships between ageing and economic growth. Hence, the Bound Test, developed by Pesaran et al. (30), has been applied to estimate the possible cointegration between variables. Model specification for the bound test is as follows:


$$\begin{array}{cc} GDPG_{t}=\;B_{0}+\overset{p}{\underset{i=1}{\sum }}B_{1i}\Delta {LAGEING}_{t-i}+\;\overset{q_{1}}{\underset{i=0}{\sum }}B_{2i}LK_{t-i}\\ + & \overset{q_{2}}{\underset{i=0}{\sum }}B_{3i}LL_{t-i}+\;\overset{q_{3}}{\underset{i=0}{\sum }}B_{4i}LHC_{t-i}+\varepsilon_{t} \end{array}$$

The selecting order of the ARDL (*p*,*q*1, *q*2, *q*3) uses the Akaike Information Criteria (AIC). The parameter containing the Σ sign indicates a short-run relationship between the variable and the dependent variable. The parameters indicate the long-run relationship between the variables in the model. Based on Pesaran et al. (30), all these variables are said to be cointegrated or possess a long-run relationship when all the variables in the above mentioned equation are simultaneously at the same level of lag. *F*-statistics from the Bound Test can be used to find the existence of cointegration among variables in the model. Commonly, the Bound Test has two critical values, known as the upper limit of I (1) and the lower limit of I (0). All variables are said to be integrated at the non-stationary level under the upper bound, whereas all the variables are integrated at the stationary under the lower bound.

These variables are said to be cointegrated and long-run relationships when the *F*-statistic value is greater than the upper bound value. An error correction model was then generated from the above ARDL model. Its analysis aims to simultaneously obtain estimated values for long and short-run parameters for all variables in the model. Lastly, these models were checked by diagnostic tests, such as the normality test, Breusch–Godfrey serial correlation LM test, Heteroskedasticity test Breusch–Pagan–Godfrey, ARCH test, cumulative sum (CUSUM), and CUSUM square test, to ensure that the models were free from unnecessary problems, such as normality, stability, serial correlation, heteroskedasticity, and misspecification.

## Results and Discussion

### Unit Root Test Result

The unit root test is used to test the stationary status of each variable, irrespective of whether the variable has unit roots. This study utilises the ADF test, which is proposed by Dickey and Fuller (31) and PP. Based on the results of the unit root test, all the variables (i.e., GDP growth, older age dependency, labour participation, physical capital, and human capital) are significant at the 1% level of significance in PP and ADF stationarity tests, at first difference. Thus, we can conclude that all the data are stationary at first difference (Table 2).

**Table 2:** Unit root test result.

**Variable**	**Augmented Dickey Fuller (ADF)**		**Philips Perron (PP)**	
	**Level**	**First difference**	**Level**	**First difference**
GDPG	−5.0765 ^***^	−7.4727^***^	−5.0765^***^	−18.7528^***^
	(0.0010)	(0.0000)	(0.0010)	(0.0000)
LAD	−3.4359	−3.8176^**^	−2.8401	−7.9575^***^
	(0.0643)	(0.0290)	(0.1929)	(0.0000)
LK	−2.1443	−5.5442^***^	−2.2664	−5.5442^***^
	(0.5056)	(0.0003)	(0.4412)	(0.0003)
LL	−4.5395^**^	−5.1811^***^	−4.5708^**^	−17.6792^***^
	(0.0044)	(0.0009)	(0.0041)	(0.0000)
LHC	−2.1334	−6.2945^***^	−2.1796	−6.2945^***^
	(0.5110)	(0.000)	(0.4864)	(0.0000)

*** and ** *denotes significant at 1 and 5% significance level, respectively. The figure in parenthesis (…) represents P-value or probability value for the significance level*.

### Estimation Output Result

In our study, the models selected for ARDL are 2, 0, 3, 4, and 4. *R*-squared results show the value of 0.9292 (which indicates the older age dependency, labour participation, physical capital, and human capital) explains nearly 92.92% of the variation in GDP growth. The remaining 7.08% is explained by other factors that are not included in this study (Table 3).

Table 3ARDL long run results.

**Table:** 

**Variable**	**Estimation of long-run coefficient**	
	**Coefficient**	***t*-Statistic**
LAD	−6.6034	−2.6792^**^
LK	3.9430	2.7213^**^
LL	33.0956	3.2736^***^
LHC	17.8709	3.4323^***^
C	−154.4565	−3.8140^***^

**Table:** 

**ARDL bound test estimate**		
*F*-statistic	11.24934	
Narayan (32)	I(0)	I(1)
**Critical value**		
10%	2.2	3.09
5%	2.56	3.49
1%	3.29	4.37

***, **, and * *are denoted as significance levels at 1, 5, and 10%, respectively*.

### Bound Testing Result

*F*-statistic in the bound test is 11.2493, which is greater than the lower bound I (0) =3.29 and upper bound I (1) = 4.37 at a 1% significant level, as obtained from the critical value table of Narayan (32). Hence, we reject the null hypothesis that no long-run relationship exists. We accept the alternative hypothesis that there is a long-run relationship between GDP growth and its determinants, which are old age dependency, labour participation, and physical and human capital. From the bound testing, there is a long-run relationship; therefore, we can further develop the ARDL model.


$$\begin{array}{c} \text{GDPG}=-154.4565-6.6034\text{LAD}+3.9430\text{LK}+33.0956\text{LL}+17.8709\text{LHC} \end{array}$$

$$\begin{array}{c} \;{\left(40.4974\right)}^{***}\;{\left(2.4647\right)}^{**}\;{\left(1.4489\right)}^{**}\;{\left(10.1098\right)}^{***}\;{\left(5.2066\right)}^{***} \end{array}$$

Based on the abovementioned equation, the result shows that there is a negative relationship between old age dependency and GDP growth. A 1% increase in old age dependency will decline GDP growth by an average of 6.6043% at the 5% level of significance. On the contrary, physical capital, labour participation, and human capital have positive relationships with GDP growth. Increasing 1% of labour participation and human capital will increase GDP growth by an average of 33.0956 and 17.8709%, respectively, and is statistically significant at the 1% level of significance. A 1% increase in physical capital will increase GDP growth by an average of 3.9430% and is statistically significant at the 5% level of significance.

The estimated result indicates that the old age dependency ratio proxies for the ageing population variables have a significant negative relationship with the economic growth in Malaysia. This implies that an increase in the ageing population will impede economic growth in Malaysia. Based on theoretical studies, Romer (17) highlighted that population ageing is favourable for long-run economic growth. However, the relative change between fertility and mortality will determine whether it is associated with an increase or decrease in long-run economic growth. This study is not aligned with the endogenous growth model by Pretnerr (20) because he argues that increases in longevity have a positive impact on economic growth. However, this result aligns with the life cycle hypothesis by Modigliani and Brumberg (13). This confirms the argument of the economic theory, which is as follows: when the population continues to age and some proportion of the population reaches their retirement age, there is a reduction in aggregate savings due to an increasingly ageing population; this situation will lead to slow economic growth.

While for empirical studies, Selvaratnam (21) and Louria (22) point out that an increase in life expectancy/an ageing population has the potential to create many economic and social issues, such as the increase in health expenditure for the aged population (23), more long-term financial resources, challenges to social security and pensions, quality of life problems, and so forth. Ismail et al. (15) also argue that the reduction in the old population dependency ratio can reduce taxes and social security amounts. This proportion of accumulated savings could have been allocated to other productive aspects of the economy. In other words, the increase in ageing might hinder economic growth.

### Error Correction Test Result

Thereafter, we examine the short-run models. We had to compute the error correction term (ECT) to form the short-run model. When we differentiate the dependent variable, it is already removed from the long-run information. Therefore, the long-run information is captured by ECT. Thus, ϕ needs to be in a negative form, −2 < ϕ < 0. The 1981–2019 result shows that the ECT was negative, which is −1.655. This indicates that the feedback mechanism is significantly effective in Malaysia. Thus, the speed of adjustment towards long-run equilibrium is 165.5% annually. Changes to the independent variables (i.e. old age dependency, labour participation, physical capital, and human capital) are corrected by GDP growth. The 165.5% deviation in the long-run was corrected by GDP growth. This reflects a significantly high-speed adjustment to long-run equilibrium after a shock. Therefore, any short-run deviation will take about 0.60 years to bring the shock back to equilibrium (Table 4).

**Table 4:** ARDL short run and diagnostic test results.

**Variable**	**Estimation of short-run coefficient**	
	**Coefficient**	***t*-statistic**
D(GDPG(-1))	0.4582	3.7118^***^
D(LK)	20.5699	10.7524^***^
D(LK(-1))	2.0003	0.6918
D(LK(-2))	11.0066	3.8146^***^
D(LL)	18.0272	3.4112^***^
D(LL(-1))	−36.4204	−5.3345^***^
D(LL(-2))	−21.3657	−3.2828^***^
D(LL(-3))	−16.7536	−3.0967^***^
D(LHC)	173.9205	1.2979
D(LHC (-1))	403.8911	2.4712^**^
D(LHC(-2))	307.5912	1.6750
D(LHC(-3))	−481.2427	−3.4383^***^
*ECT*_*t*−1_	−1.6550	−9.3460^***^
**Diagnostic checking**		
**Test**		***P*** **-value**
Normality		0.7136
BG serial correlation LM		0.4166
Heteroskedasticity		0.4364
ARCH		0.1768

***, **, and * *are denoted as significance levels at 1, 5, and 10%, respectively*.

## Diagnostic Check Test Result

### Histogram-Normality Test

Based on the Histogram-normality test, it seems that the residuals are normally distributed as the Jarque-Bera value of 0.6750 with a *P*-value > 5% significance level. Therefore, it indicates that the data have residual or error terms which are normally distributed; the error term follows the normal distribution.

### Serial Correlation Test

For the serial correlation test, we used the Breusch–Godfrey serial correlation LM test. We found that there is a higher order correlation between error terms. The test shows that the *P*-value (0.4166) is > 5% significance level. Thus, we fail to reject the null hypothesis of no autocorrelation. A higher probability value > 0.05 strongly indicates the absence of a serial correlation in the residuals. Thus, we can conclude that the data do not have an autocorrelation problem.

### Heteroskedasticity Test

For the heteroscedasticity test, we used the Breusch–Pagan–Godfrey Heteroskedasticity test result. Under the null hypothesis, there is an absence of the heteroskedasticity problem and the presence of homoscedasticity. The variance of the error term is constant. For the alternative hypothesis, there is a presence of the heteroscedasticity problem and an absence of homoscedasticity. This test found that the *P*-value (0.4364) is >5% significance level. Therefore, we fail to reject the null hypothesis of no pure heteroscedasticity. We can conclude that there is no heteroscedasticity problem and the error terms have constant variance.

### Autoregressive Conditional Heteroskedasticity

Based on the ARCH result, the *P*-value (0.1768) is more than the 5% significance level. Thus, we fail to reject the null hypothesis of no ARCH problem; therefore, our data do not have ARCH problems.

### Stability Test

**Figure 2:**
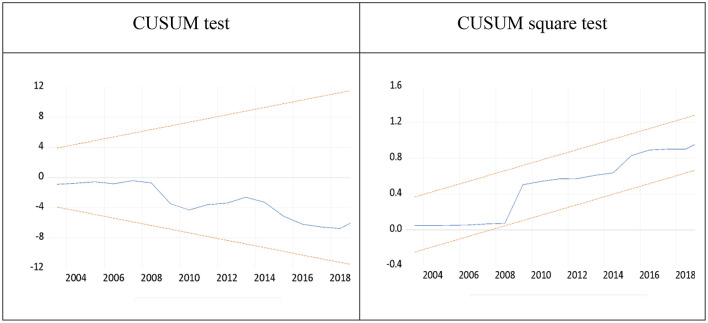
CUSUM and CUSUM square test results.

Based on Figure 2, a visual inspection of the plot above indicates that the plots of CUSUM appear inside 5% critical bands or stay within the critical bounds. These simply imply that the estimated parameters in the CUSUM test are stable over the sample period. Similarly, the estimated parameters in the CUSUM square test are stable or stationary across our period of study.

## Conclusion and Policy Recommendations

This study attempts to investigate the impact of the ageing population on the economic growth for the short- and long-run estimations in Malaysia by using time series data. Its estimated result indicates that the old age dependency ratio proxies for the ageing population variables have a significant negative relationship to economic growth in Malaysia. This implies that an increase in the ageing population will impede economic growth in Malaysia. Although control variables (such as physical capital, labour participation, and human capital) have positive relationships with economic growth in Malaysia, there is evidence of a long- and short-run relationship between economic growth and ageing population variables, and also control variables.

Romer (17) highlighted that population ageing is favourable for long-run economic growth. However, the relative change between fertility and mortality will determine whether it is associated with an increase or decrease in long-run economic growth. This study is not aligned with the endogenous growth model by Pretnerr (20) because he argues that increases in longevity have a positive impact on economic growth. However, this result aligns with the life cycle hypothesis by Modigliani and Brumberg (13). This confirms the argument of the economic theory, which is as follows: when the population continues to age and some proportion of the population reaches their retirement age, there is a reduction in aggregate savings due to an increasingly ageing population; this situation will lead to slow economic growth. Moreover, Selvaratnam (21) and Louria (22) point out that an increase in life expectancy/an ageing population has the potential to create many economic and social issues, such as the increase in health expenditure for the aged population (23), more long-term financial resources, challenges to social security and pensions, quality of life problems, and so forth. Ismail et al. (15) also argue that the reduction in the old population dependency ratio can reduce taxes and social security amounts. This proportion of accumulated savings could have been allocated to other productive aspects of the economy. In other words, the increase in ageing might hinder economic growth. Furthermore, the findings in this study are in tandem with the findings of Brendan and Sek (24), Maestas et al. (12)^4^, Teixeira et al. (26), and Rahman (25).

Based on our results, we found that labour participation and human capital are the most significant determinants of GDP growth in Malaysia. This finding confirms that the endogenous growth theory (which is labour productivity and human capital) is another growth engine, along with physical capital. Thus, we recommend that policymakers and economic planning authorities control these two variables to boost the growth of the economy so that Malaysia can become a developed country by 2030. These variables should be reviewed along with related mechanisms to achieve higher productivity, sustainable economic development, and growth.

Furthermore, Malaysian policymakers and authorities should consider active ageing policies that are widely implemented in Japan, such as the Silver Human Resource Centre, that employ the elderly into the workforce because this group has a lot of experience and can contribute to ideas and guidance for future generations. Recently, there is a rising trend of an ageing population in Malaysia. This raises concerns about whether Malaysia will suffer from the negative effect of old age, decreasing saving rates, and possible unemployment in the future. Indubitably, all these issues require further analyses and are on our future research agenda.

## Data Availability Statement

Publicly available datasets were analysed in this study. This data can be found at: the world bank and penn world table.

## Author Contributions

AI and SM contributed to the conception and design of the study, organised the database, performed the statistical analysis, and wrote the first draft of the manuscript. All authors contributed to manuscript revision, read, and approved the submitted version.

## Funding

This work was supported by Universiti Kebangsaan Malaysia grant code EP-2018-001.

## Conflict of Interest

The authors declare that the research was conducted in the absence of any commercial or financial relationships that could be construed as a potential conflict of interest.

## Publisher's Note

All claims expressed in this article are solely those of the authors and do not necessarily represent those of their affiliated organizations, or those of the publisher, the editors and the reviewers. Any product that may be evaluated in this article, or claim that may be made by its manufacturer, is not guaranteed or endorsed by the publisher.

## Footnotes

^1^ https://www.who.int/news-room/q-a-detail/population-ageing

^2^ http://www.nber.org/papers/w22452.pdf

^3^ http://www.nber.org/papers/w22452.pdf

^4^ http://www.nber.org/papers/w22452.pdf

## References

[1] WHO. Ageing: Global Population. World Health Organization. (2010, October 2). Available online at: https://www.who.int/news-room/q-a-detail/population-ageing (accessed April 11, 2021).

[2] DOSM. Current Population Estimates, Malaysia, 2020. Department of Statistics Malaysia (2020, July 15).

[3] World Bank. Ageing Dependency Ratio. World Development Indicator (2021). Retrieved from: https://databank.worldbank.org/source/world-development-indicators

[4] World Bank. Fertility Rate. World Development Indicator (2021). Retrieved from: https://databank.worldbank.org/source/world-development-indicators

[5] World Bank. Government Health Expenditure (% of GDP). World Development Indicator (2021). Retrieved from: https://databank.worldbank.org/source/world-development-indicators

[6] Tang CF Tey NP. Low fertility in Malaysia: can it be explained? J Pop Res. (2017) 34:101–18. 10.1007/s12546-017-9187-2

[7] Otsu K Shibayama K. Population aging and potential growth in Asia. Asian Dev Rev. (2016) 33:56–73. 10.1162/ADEV_a_00072

[8] de Meijer C Wouterse B Polder J Koopmanschap M. The effect of population aging on health expenditure growth: a critical review. Eur J Ageing. (2013) 10:353–61. 10.1007/s10433-013-0280-x 28804308 PMC5549212

[9] Elsawahli HMH Ahmad FB Ali AS. Demographic transition and sustainable communities in Malaysia. Plann Malay J. (2016) 14:39–48. 10.21837/PMJOURNAL.V14.I5.19134614000

[10] D'Ambrogio E. Japan's Ageing Society. European Union: European Parliamentary Research Service (2020).

[11] Börsch-Supan A Hunklerad C Weissaef M. Big data at work: age and labor productivity in the service sector. J Econ Ageing. (2021) 19:100319. 10.1016/j.jeoa.2021.100319

[12] Maestas N Mullen KJ Powell D. The Effect of Population Ageing on Economic Growth, the Labor Force and Productivity. National Bureau of Economic Research Working Paper Series. (2016). Available online at: http://www.nber.org/papers/w22452.pdf (accessed April 15, 2021).

[13] Modigliani F Brumberg R. Utility analysis and the consumption function. In: Kurihara K, editor. Post-Keynesian Economics, New Brunswick: Rutgers University Press. (1954). p. 151–70.

[14] Gruescu S. Population Ageing and Economic Growth. Education Policy and Family policy in Model of Endogenous Growth. Contribution to Economics. Hiedelberg: Physica-Verlag HD (2007).

[15] Ismail N Rahman HSWHA Hamid TATA Said R. Aging and economic growth: empirical analysis using autoregressive distributed lag approach. Sains Malays. (2016) 45:1345–50.

[16] Malthus T. An Essay on the Principle of Population. London: J. Johnson (1798).

[17] Romer PM. Endogenous technological change. J Polit Econ. (1990) 98:S71–102.

[18] Grossman GM Helpman E. Quality ladders in the theory of economic growth. Rev Econ Stud. (1991) 58:43–61.

[19] Aghion P Howitt P. A model of growth through creative destruction. Econometrical. (1992) 60:323–51.

[20] Prettner K. Population aging and endogenous economic growth. J Popul Econ. (2013) 26:811–34. 10.1007/s00148-012-0441-9 23576847 PMC3617593

[21] Selvaratnam DP Idris NA Bakar NA Kim OB. The effects of increased life expectancy in Malaysia. Prosiding Perkem IV. (2009) 1:305–15.

[22] Louria DB. Extraordinary longevity: individual and societal issues. J Am Geriatr Soc. (2005) 53(9 Suppl):S317–9. 10.1111/j.1532-5415.2005.53499.x 16131362

[23] Baharin R Saad S. Ageing population and health care expenditure: evidence using time series analysis. Malay J Soc Space. (2019) 14:65–73. 10.17576/geo-2018-1404-0612563659

[24] Brendan LR Sek SK. The relationship between population ageing and the economic growth in Asia. Adv Indust Appl Math. (2016). 1750:060009. 10.1063/1.495461424313031

[25] Rahman SW. Impact of Population Aging on Economic Growth, Health Expenditure and Labor Productivity in Malaysia. School of Graduates Studies, Universiti Putra Malaysia (UPM) (2018).

[26] Teixeira A Nagarajan R Silva S. The impact of ageing and the speed of ageing on the economic growth of least developed, emerging and developed countries, 1990–2013. Rev. Dev Econ. (2017). 21:909–34. 10.1111/rode.12294

[27] Taasim S. Ageing population and economic growth: evidence from Malaysia. South Asian J Soc Stud Econ. (2020). 7:11–8. 10.9734/sajsse/2020/v7i430196

[28] Futagami K Nakajima T. Population aging and economic growth. J Macroecon. (2013). 23:31–44. 10.1016/S0164-0704(01)00153-7

[29] Scarth W. Population ageing, productivity and living standards. In: Sharpe A St-Hilaire F Banting K, editors. The Review of Economic Performance and Social Progress: Towards a Social Understanding of Productivity. Montreal, QC: IRPP (2002). p. 145–56.

[30] Pesaran MH Shin Y Smith RJ. Bound testing approaches to the analysis of level relationships. J Appl Econ. (2001). 16:289–326. 10.1002/jae.61

[31] Dickey DA Fuller WA. Likelihood ratio statistics for autoregressive time series with a unit root. Econometrica. (1981) 49:1057–72.

[32] Narayan PK. Reformulating Critical Values or the Bound F-Statistics Approach to Cointegration: An Application to the Tourism Demand Model for Fiji. Department of Economics Discussion Papers No. 02/04. Melbourne, Australia: Monash University (2005).

